# FOXO3a and Its Regulators in Prostate Cancer

**DOI:** 10.3390/ijms222212530

**Published:** 2021-11-20

**Authors:** Dominika Ewa Habrowska-Górczyńska, Marta Justyna Kozieł, Karolina Kowalska, Agnieszka Wanda Piastowska-Ciesielska

**Affiliations:** Department of Cell Cultures and Genomic Analysis, Medical University of Lodz, Zeligowskiego 7/9, 90-752 Łódź, Poland; dominika.habrowska@umed.lodz.pl (D.E.H.-G.); marta.koziel@umed.lodz.pl (M.J.K.); karolina.kowalska1@umed.lodz.pl (K.K.)

**Keywords:** prostate cancer, foxo3a, Akt, proliferation, apoptosis

## Abstract

Forkhead box O3 (FOXO3a) is a member of a subfamily of forkhead transcription factors involved in the basic processes within a cell, including proliferation, apoptosis, cell cycle regulation, and DNA damage. As a transcription factor, FOXO3a is involved in the response to cellular stress, UV radiation, or oxidative stress. Its regulation is based on the modification of proteins as well as regulation by other proteins, e.g., growth factors. FOXO3a is commonly deregulated in cancer cells, and its inactivation is associated with initiation and progression of tumorigenesis, suggesting its role as a tumor suppressor; however, its role is still disputed and seems to be dependent on upstream signaling. Nevertheless, FOXO3a serves as an interesting potential target in therapies as it is regulated during treatment with very common anti-cancer drugs such as paclitaxel, cisplatin, docetaxel, and doxorubicin. This review aims to update the reported role of FOXO3a in prostate cancer (PCa), with a focus on its regulators that might serve as potential therapeutic agents in PCa therapy.

## 1. Introduction

Prostate cancer (PCa) is one of the most common malignancies in men in Europe and the United States of America (USA) [1,2]. The increasing survival of patients is possibly associated with the exploitation of prostate-specific antigen (PSA), which is currently used as a prognostic marker and is detectable in men’s serum [3]. Age, heredity, race, and lifestyle are known to be risk factors for PCa development [4]. PCa is an endocrine-dependent disease in which androgens play a crucial role; thus, androgen-deprivation therapy (ADT) and androgen receptor (AR) targeted therapy are gold standards in combination treatment (e.g., radiotherapy + ADT) or in palliative care [5]. These therapies work satisfactorily in androgen-dependent PCa; however, following ADT and as the disease progresses and metastasizes, most patients gradually develop an androgen-independent state. Initially, androgen-dependent PCa becomes castration-resistant PCa (CRPC), which carries poor prognosis and leaves limited treatment options [4]. It was reported that failure of ADT is a cause of approximately 27,000 deaths caused by PCa metastases in the USA [6]. On a molecular level, the tumor becomes more aggressive through adaptive responses and relies more on other molecular pathways involved in the regulation of proliferation and/or apoptosis to overcome the blockage of ARs [7]. The efficacy of the available therapy options is poor, with a median survival benefit of 2-5 months. Thus, there is a high demand for novel treatment modalities that offer both high efficacy and minimal side effects, which seems to be challenging in chemotherapy [8].

Phosphatase and tensin homolog (PTEN) mutation is one of the most common genetic alterations in PCa associated with a Gleason Score as well as poor prognosis. It is estimated that a loss of PTEN function by mutation or deletion is present in almost 50% of CRPC cases [9]. Tumor suppressor PTEN blocks constitutive activation of the Phosphoinositide 3-kinases (PI3K)/protein kinase B (Akt)/mammalian target of the rapamycin (mTOR) signaling pathway, which regulates cell proliferation, migration, apoptosis, autophagy, etc. [10]. The PI3K/Akt/mTOR signaling pathway is also reported to participate in genomic and non-genomic AR signaling as well as in the regulation of transcription factors, e.g., FOXO3a. FOXO3a is identified as an important player in the development and pathology of PCa, so therapeutics that modulate FOXO3a activity might serve as interesting novel agents in PCa treatment and prevention [7]. Therefore, in this review, we summarize the knowledge concerning the reported modulators of FOXO3a in PCa with a special focus on miRNA, which represents an interesting therapeutic option for PCa.

## 2. FOXO3a Structure, Function, and Regulation of Activity

**Figure 1:**
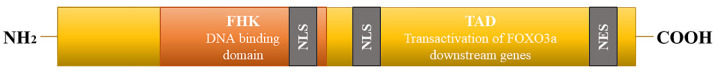
Structure of the FOXO3 protein. FHK: forkhead winged helix-turn-helix DNA binding domain; NLS: nuclear localization sequence; TAD- C: C-terminal transactivation domain; NES: nuclear export sequence.

Forkhead box O3, also named FOXO3 or FOXO3a, belongs to the forkhead box (FOXO) family in which four isoforms may be distinguished: FOXO1, FOXO2, FOXO3a, and FOXO4. The FOXO3a gene is located on chromosome 6 [11]. The protein product has a mass of about 71 kDa and consists of five major domains: a highly conserved forkhead winged helix-turn-helix DNA binding domain (FKH) responsible for the interaction between FOXO3a and DNA; two nuclear localization sequences (NLS) involved in the repositioning of the FOXO3a factor (from the cytoplasm to the nucleus and vice versa); a nuclear export sequence (NES); and a C-terminal transactivation domain (TAD), which is fundamental for the transactivation of FOXO3a downstream genes (Figure 1) [12]. Moreover, it was reported that the FKH region is responsible for interactions with estrogen receptor α (ERα) and p53 [13]. Physiologically, the FOXO family is involved in the regulation of diverse cellular physiological events such as apoptosis [14], cell cycle progression [15], DNA damage [16], proliferation [17], and response to oxidative stress [18,19]. Because of the multiple functions of this transcription factor, dysregulation of its expression is associated with many disorders, including cancers and other non-neoplastic diseases [12,20]. For example, FOXO3a was observed to be affected in the neurodegeneration process in Alzheimer’s disease [21], while in patients with hepatocellular carcinoma, its expression is significantly higher than in healthy patients [22]. The loss of FOXO3a was reported to induce PCa progression in the androgen-independent type of PCa [23]. In PCa patients, an increased cytoplasmic expression of the phosphorylated form of FOXO3a (Ser253) was observed, which in turn correlated with disease progression (Gleason score) in contrast to benign prostate cells [24]. Overexpression of FOXO3a in prostate cells was reported to activate apoptotic signaling and to affect cell proliferation, suggesting that FOXO3a might act as an interesting agent due to being involved in the activation of pro-survival agents as well as proapoptotic agents via oxidative stress in PCa cells [25].

**Figure 2:**
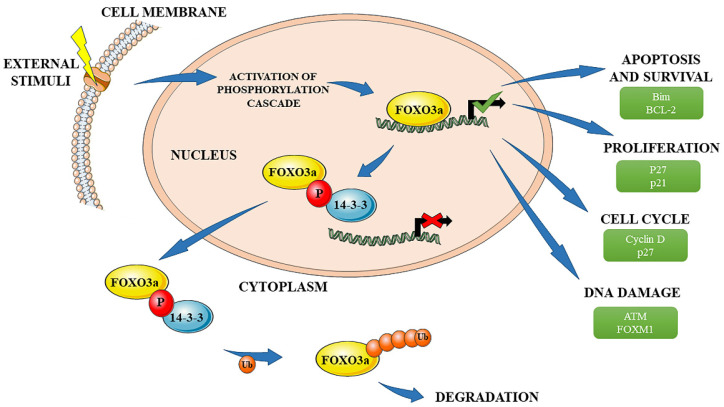
Schematic diagram showing the activity of FOXO3a transcription factor and its role in cells with examples of genes associated with particular processes. The graphical illustration was prepared by using the images from Servier Medical Art by Servier (https://smart.servier.com/smart_image/, accessed on 10 November 2021).

Naturally, FOXO3a is found in the nucleus, where it is bound up with DNA and actively regulates the expression of genes involved in multiple cellular events. After phosphorylation (by PKB, ERK, SGK, or IKKβ), FOXO3a changes its conformation, binds with 14-3-3 protein, and then translocates to the cytosol, where it is degraded (Figure 2). Because of the fact that FOXO3a acts as a tumor suppressor, its degradation leads to increased proliferation, cell transformation, and decreased apoptosis, among others. Therefore, regulation of its expression and localization plays a crucial role in maintaining homeostasis in the body. Multiple factors and processes are involved in the control of its expression. Micro-ribonucleic acids (miRNAs), phosphorylation, acetylation/deacetylation, and a number of other posttranslational modifications control the regulation of FOXO proteins, while external stimuli are responsible for protein expression, cellular localization, DNA binding efficiency, and transactivation of downstream genes [12,26]. The broad group of stimuli that may affect the activity of the FOXO3a factor includes, inter alia, epidermal growth factor receptors (EGFR), insulin, nutrients, and various molecules involved in the response to oxidative stress. Many regulatory pathways have been identified as being implicated in the regulation of FOXO3a. In PCa, it was observed that mitochondrial NAD-dependent protein deacetylase sirtuin-3 (SIRT3) modulates epithelial to mesenchymal transition (EMT) and migration via attenuating the WNT/β-catenin pathway and thus leads to upregulated expression of FOXO3a [27]. The relationship between FOXO3a and the WNT/β-catenin pathway, and their effect on EMT was also observed by other researchers [28]. The Akt/PBK pathway was reported to negatively regulate FOXO3a activity [24], while estrogen receptor β (ERβ) positively regulates FOXO3a and induces apoptosis via p53 upregulated modulator of apoptosis (PUMA) [29]. The influence of the phosphoinositide 3-kinases (PI3K), extracellular signal-regulated kinases (ERK), and mitogen-activated protein kinases (MAPK) signaling pathways on FOXO3a were also presented [30,31,32].

## 3. FOXO3a Modulators

Numerous research studies have documented that the most potent regulators of FOXO3a in PCa, among others, are dietary agents such as apigenin, sulforaphane, or 3,3′-Diindolylmethane. Therefore, we decided to review the mechanisms behind those compounds with respect to their activity associated with FOXO3a in prostate cancer. 

### 3.1. Apigenin

Apigenin—a flavonoid present in vegetables, fruits, herbs, and spices—is known to possess anti-inflammatory and antioxidant properties [33]. In the mouse model, apigenin was reported to inhibit the progression of prostate carcinogenesis via a reduction in both the genitourinary apparatus as well as the dorsolateral and ventral prostate, as compared to the control group not receiving apigenin for 20 weeks. Moreover, the regulation of differentiation of prostate tissue was observed. After treatment with apigenin, the number of the well-differentiated, moderately differentiated, and poorly differentiated cancers decreased. An increase in the expression of FOXO3a in the nucleus of prostate cells was detected, with a simultaneous decrease in the phosphorylation of FOXO3a at Ser253 and Akt at Ser473. At once, it was followed by an increased DNA binding affinity. The observed nuclear translocation and DNA binding of FOXO3 were associated with downstream targets of FOXO3a: Bcl-2-like protein 11 (Bim), p27, and cyclin D1. The increased DNA binding was also confirmed in an in vitro study on the PCa cancer cell lines LNCaP and PC-3, where an increased binding of FOXO3a to p27, in turn, caused decreased proliferation and cell cycle arrest in the G0/G1 cell cycle phase [30]. 

### 3.2. Resveratrol

Another flavonoid, resveratrol, was reported to affect prostate carcinogenesis. Resveratrol (3,4′,5-trihydroxystilbene), a phytoalexin commonly found in many plants, possesses a documented anti-inflammatory and antioxidative effect and is believed to be a cause of benefits associated with the Mediterranean diet. First, resveratrol was reported to reduce cell viability and to induce apoptosis of benign prostate hyperplasia (BPH) cells, in which downregulation of FOXO3a and p38 MAPK activation were involved in the observed apoptosis. The beneficial role of resveratrol alone and in combination therapy with tumor necrosis factor (TNF)-related apoptosis-inducing ligand (TRAIL) was also reported. In PC-3 mice xenografts, resveratrol enhanced TRAIL-induced apoptosis associated with cell cycle regulatory proteins cyclin D1 and p27. The research also confirmed previous observations made by Cheng et al. that the activation of FOXO3a causes an upregulation of Bim, TRAIL, death receptors 4 and 5 (DR4 and DR5), and p27/Kip1 and triggers apoptosis in LNCaP prostate cancer cells [34]. Furthermore, the combination treatment with resveratrol and TRAIL resulted in the inhibition of the phosphorylation of FOXO3a and enhanced its binding with DNA, which in consequence triggered lower metastases and angiogenesis in PC3-mice xenografts [35].

### 3.3. Diosmetin

Another plant flavonoid, diosmetin, has been shown to selectively induce apoptosis and to inhibit the growth of cancer cells [36]. In PCa cells, diosmetin reduced cell growth and caused cell cycle arrest in the G0/G1 cell cycle phase with decreased expression of cyclin D1 and cyclin E, as well as cyclin-dependent kinase (cdk2) and cyclin-dependent kinase 4 (cdk4); moreover, it increased the expression of p27/Kip1. The reduced growth of cells was associated with the induction of apoptosis via the regulation of bcl-2 associated X protein (Bax), B-cell lymphoma 2 (Bcl-2), cleaved caspase-3, and cleaved poly (ADP-ribose) polymerase (PARP) expression. Regulation of c-Myc/FOXO3a/p27/Kip1 was proposed as a molecular mechanism of diosmetin-induced apoptosis due to the observed decreased expression of c-Myc and increased expression of FOXO3a and its downstream target p27/Kip1 [36].

### 3.4. Sulforaphane

Another natural compound, sulforaphane (SFN), found in Cruciferous vegetables was observed to have antioxidant, anti-proliferative, and anti-carcinogenic properties in prostate cells [37]. On the molecular level, SFN induces oxidative stress in cells, increases the level of Fas ligand (FasL), activates caspase 8 and cleavage of BH3-interacting domain death agonist (Bid), and in consequence triggers apoptosis in androgen-independent prostate cancer cells (PC-3 and DU-145) [37]. Similar to previous research, SFN in a combination treatment with TRAIL enhanced the effect of such a therapeutic intervention in vitro in PC-3 cells and TRAIL-resistant LNCaP cells. In the in vivo model, SFN suppressed the phosphorylation of Akt; the extracellular signal-regulated kinase 1/2 (ERK1/2); FOXO3a; and p65, a subunit of the NF-kappa-B transcription complex (p65-NFκB). Moreover, SFN alone inhibited FOXO3a phosphorylation, and the results from an in vivo study suggested that SFN activates FOXO3a through the dephosphorylation of Akt and ERK1/2 and may regulate FOXO-dependent gene transcription and apoptosis [38].

### 3.5. 3,3′-Diindolylmethane

3,3′-Diindolylmethane (DIM)—a compound derived from the digestion of cruciferous vegetables—was also found to induce apoptosis in PCa cells [39]. Li et al. reported that formulated DIM (B-DIM) decreased the phosphorylation of FOXO3a and showed that B-DIM regulates FOXO3a phosphorylation, not its nuclear importing. Moreover, the authors showed that B-DIM caused binding of FOXO3a to p27/KIP1 and the AR promoter, which resulted in an increase in the expression of p27/KIP1 and decreased the expression of ARs in PCa cells. B-DIM also showed downregulation of the WNT signaling pathway and inhibited the survival of cells via the glycogen synthase kinase 3 beta (GSK-3β)/β-catenin signaling pathway, in which FOXO3a also participates. The authors suggested that the Akt/FOXO3a/GSK-3β/β-catenin/AR signaling pathway is responsible for the observed inhibition of proliferation of PCa cells [40]. 

### 3.6. Platycodin D

Platycodin D (PD), derived from the plant *Platycodin grandiflorum*, presented cytotoxic properties against PCa cells, with no effect on normal prostate RWPE-1 cells. Zhou et al. observed that PD decreased the proliferation of PC-3, DU-145, and LNCaP cells and induced apoptosis and cell cycle arrest in the G0/G1 or G2/M cell cycle phases in the case of PC-3 cells. The expression of FOXO3a increased, whereas its phosphorylation decreased. Similarly, a decreased expression of mouse double minute 2 homolog (MDM2) was also observed. Downstream targets of FOXO3a, p21 and p27, were upregulated. MDM2 silencing resulted in an increased FOXO3a expression, whereas MDM2 overexpression triggered a contradictory effect. The results from in vitro assays were confirmed in an in vivo model with BALB/c nude mice PC-3 xenografts [41].

### 3.7. β-Arrestin 1

The interaction between FOXO3a and MDM2 was also suggested in another study. Kong et al. showed that β-arrestin 1, a negative regulator of G-protein-coupled receptors (GPCR) that plays a role in the regulation of proliferation and apoptosis in cells, might promote PCa cell growth via the inhibition of FOXO3a [42]. The authors showed that the lack of β-arrestin 1 in PCa cells decreases cell growth and invasion. The expression of FOXO3a was negatively correlated with the expression of β-arrestin 1 in normal and PCa cells. Moreover, the ectopic expression of β-arrestin 1 increased the ubiquitination of FOXO3a via the formation of a complex with FOXO3 and MDM2 and accelerated the interaction between FOXO3a and MDM2 [42]. 

### 3.8. Non-Dietary Agents

Besides natural substances, other chemical compounds were suggested to affect PCa by targeting FOXO3a. NSC126188, a piperazine alkyl derivative, induced apoptosis in PC-3 cells by activation of caspase 3, cleavage of PARP, and increased p21 expression. On a molecular level, the authors showed that NSC126188 might act as an inhibitor of Akt phosphorylation at S473 and T308. Moreover, the dephosphorylation of FOXO3a at S253 and Bcl-2 associated death promoter (Bad) at S99 was associated with the observed induction of apoptosis. It was also suggested that FOXO3a functions as a transcription factor in the presence of NSC126188 due to the decreased phosphorylation of FOXO3a in the cytoplasm of cells and increased dephosphorylation in the nuclei. Moreover, the increased expression of FOXO3a-targeted genes p21, p27, and FasL was also observed. The involvement of FOXO3a in observed apoptosis in PCa cells was confirmed by FOXO3a knockout with siRNA. FOXO3a-negative cells demonstrated reduced activation of caspase-3 and PARP cleavage and, in consequence, a reduced ratio of apoptosis [43].

Valproic acid (VPA), used as an anti-epileptic drug, was demonstrated to induce cell cycle arrest determined by the modulation of p21 and p27 as well as cyclin D1 in an in vivo model of PCa. Decreased tumor growth in a xenograft model was associated with the induction of apoptosis in cells [44]. Although the authors did not evaluate the modulation of FOXO3a, the observed effect as well as activation of p27 suggest the role of FOXO3a in the induction of apoptosis by VPA. Another study showed that VPA in LNCaP cells reduced proliferation and modulated progression of the cell cycle; however, the authors suggested that VPA induced phosphorylation of Akt, ERK1/2, and mTOR signaling as well as upregulation of FOXO3a and, in consequence, reduced tumor suppressor activity. VPA caused reduced expression of ARs and PSA but altered LNCaP cell morphology, which in consequence led to neuroendocrine differentiation confirmed with β-III Tubulin and γ-Enolase protein levels. Thus, it appears that, although VPA reduces proliferation of PCa cells, it might counteract neuroendocrine differentiation related to advanced disease and poor prognosis for PCa patients [45]. A summary of information for all of the above-mentioned compounds is provided in Table 1.

## 4. miRNAs as Regulators of FOXO3 in PCa

miRNAs are small (18-25 nucleotides), non-coding, single-stranded RNA molecules [46]. They are responsible for the regulation of almost all biological processes in cells via controlling the expression of various genes (they modify stability and translation of mRNA post-transcriptionally) [47]. Similar to the case of FOXO3a, dysregulation of miRNAs contributes to pathological conditions in organisms, which in consequence leads to many diseases. Disturbances in the expression (both overexpression and underexpression) of miRNA molecules are observed in almost every cancer [48,49,50,51]. Furthermore, it has been reported that they may also affect response to treatment [52,53]. Therefore, they are undoubtedly considered critical factors in human carcinogenesis. Recently, a rapidly increasing number of studies have proposed that miRNAs may have a beneficial impact on cancer treatment and diagnosis. In prostate cancer, many molecules have been investigated; however, their association with the FOXO3a factor is poorly elucidated [54]. Nevertheless, several studies shed new light on this issue. Feng et al. showed that miR-223-3p may increase the chemosensitivity of prostate cancer cells in both in vitro and in vivo studies [55]. On the contrary, Zhou et al. noted that miR-223-3p decreased radiosensitivity via targeting FOXO3a [56]. In DU-145 cells, it was observed that miR-592 regulates growth and proliferation via targeting FOXO3a agents [57]. miR-1307 overexpression leads to decreased expression of FOXO3a and thus to increased proliferation and tumorigenesis [58]. Chen et al. showed that miR-590-3p influences the proliferation and invasion of LNCaP cells and that this effect is partially regulated by the Akt/FOXO3a pathway [59]. MiR-96 was observed to be upregulated in prostate cancer [60]. Along with the higher expression of miR-96, the expression of FOXO3a decreases and proliferation increases [60]. Huang et al. noticed that miR-197-3p may reduce proliferation in PCa cells and that its overexpression stimulates FOXO3a expression [61]. Nevertheless, the knowledge about particular miRNA molecules and their association with FOXO3a and the prostate gland is limited. Therefore, more studies should be carried out to improve present treatment options. Summarized information is presented in Table 2.

## 5. Conclusions

Over the past two decades, medicine has made significant progress in the field of cancer management. Nevertheless, treating cancers, especially those diagnosed at a late stage, remains a major challenge. Therefore, many alternative treatment modalities are being tested to improve the currently available therapeutic strategies. Understanding the molecular pathways related to the progression and/or response to chemotherapy of PCa is crucial for new drug design. FOXO3a appears to be a promising target in many types of cancer, including prostate cancer. Numerous compounds, both natural and chemical, cited in this review regulate the behavior of prostate cancer cells via the modulation of FOXO3a expression and activity, underlining its importance in this disease. It is worth noting that more research studies concerning the FOXO3a factor as a target in cancer therapy may shed new light on cancer treatment and therefore bring better therapeutic outcomes.

## Figures and Tables

**Table 1:** Summarized information related to compounds that regulate FOXO3a expression.

Substance	Experiment Model	Involved Pathways	FOXO3a	Effects	References
Apigenin	in vivo (TRAMP mice), in vitro (LNCaP, PC-3)	PI3K/Akt/FoxO signaling pathway	↑ activity	Tumor growth suppression, reduced proliferation, G1-phase arrest	[30]
β-arrestin1	in vivo (nude mice), in vitro (RWPE-1, LNCaP, C4-2, PC-3 and DU-145)	MDM2-mediated ubiquitylation pathway	↓ expression ↑ degradation	PCa growth, promote the cell growth of CRPC cells	[42]
NSC126188	in vitro (PC-3)	Akt signaling	↑ dephosphorylation of FOXO3a	Apoptosis induction	[43]
Platycodin D	in vivo (BALB/c nu/nu mice), in vitro (DU-145, PC-3, LNCaP)	MDM2-mediated ubiquitylation pathway	↑ protein level	Cell cycle arrest, apoptosis induction	[41]
Resveratrol	in vitro (BPH-1)	p38 MAPK	↓ protein expression	ROS accumulation, apoptosis induction	[32,35]
Diosmetin	in vitro (LNCaP, PC-3)	c-Myc decrease	↑ protein expression	Cell growth inhibition and apoptosis	[36]
Valproic Acid	in vitro (LNCaP)	Akt, ERK1/2	↓ protein level	Maintaining cell tumorigenesis	[45]
Sulforaphane	in vivo (BALB/c nu/nu mice), in-vitro (LNCaP, PC-3)	Ras/MEK/ERK and PI3K/AKT pathways	inhibited phosphorylation of FOXO3A	Inhibition of angiogenesis, induction of apoptosis	[38]
3,3’-Diindolylmethane	in vitro (LNCaP, C4-2B)	Akt/FOXO3a/GSK-3β/β-catenin/AR signaling	↓ phosphorylation ↓ the ratio of p-FOXO3a over FOXO3a in both the cytoplasm and nucleus	Inhibited cell proliferation and induced apoptotic cell death	[40]

**Table 2:** Summarized information about miRNA and their influence on FOXO3 expression and the effect in cells.

miRNA	Experimental Model	FOXO3a Expression	Effects	References
miR-223-3p	in vitro (C4-2, LNCaP, PC-3, DU-145, RWPE-1), in vivo (BALB/c nude mice)	↑	Increased chemosensitivity to docetaxel	[55]
miR-223-3p	in vitro (C4-2, LNCaP, PC-3, DU-145, RWPE-1)	↓	Increased radiotherapy resistance	[56]
miR-592	in vitro (M12, Tsu-Pr1, PC-3, DU-145, 22RV1, LNCAP, RWPE-1)	↓	Increased growth and proliferation	[57]
miR-1307	in vitro (Tsu-Pr1, DU-145, PC-3, LNCAP and 22RV1)	↓	Increased proliferation and tumorigenesis	[58]
miR-590-3p	in vitro (LNCaP, 22RV1, VCaP, C4-2, PC-3, DU-145, RWPE-1)	↑	Increased proliferation and invasion	[59]
miR-96	in vitro (PC-3)	↓	Increased proliferation	[60]
miR-197-3p	in vitro (C4-2, DU145, 22Rv1)	↑	Suppressed growth	[61]

## Data Availability

Data available upon request.
